# The Reliability and Validity of a Self-Report Measure of Cognitive Abilities in Older Adults: More Personality than Cognitive Function

**DOI:** 10.3390/jintelligence6010001

**Published:** 2017-12-26

**Authors:** Danielle Herreen, Ian Taylor Zajac

**Affiliations:** 1 School of Psychology, University of Adelaide, Adelaide 5000, Australia; danielle.herreen@csiro.au; 2 Health & Biosecurity, Commonwealth Scientific and Industrial Research Organisation (CSIRO), Adelaide 5000, Australia

**Keywords:** cognitive function, cognitive ageing, self-report, scale development, personality

## Abstract

The development of brief, reliable and valid self-report measures of cognitive abilities would facilitate research in areas including cognitive ageing. This is due to both practical and economic limitations of formal cognitive testing procedures. This study examined the reliability and validity of the newly developed Self-Report Measure of Cognitive Abilities (SRMCA; Jacobs & Roodenburg, 2014); a multi-item self-report tool designed to assess cognitive function in the ability areas of fluid reasoning (Gf), comprehension-knowledge (Gc) and visual processing (Gv). Participants were (*n* = 93) cognitively healthy older adults aged between 52 and 82 years who completed the SRMCA, the Big Five Inventory and a battery of cognitive tasks. Results revealed adequate reliability for the SRMCA and convergent validity for the Gc domain but not for Gf or Gv. Moreover, significant personality bias was evident with Extraversion (positively), Openness to Experience (positively) and Neuroticism (negatively) predicting SRMCA responses independently of actual cognitive performance. Thus, although the SRMCA appears to be reliable in older adults, personality was a stronger predictor of self-estimated cognitive abilities than actual cognitive performance, questioning the utility of this tool as a subjective measure of cognitive ability.

## 1. Introduction

Cognitive function is one of the most widely studied topics in Psychology and interest in human intellectual function predates the modern tests with which it is measured. Plato and Aristotle, for example, were amongst the first to discuss the heritability of intelligence [1], an idea subsequently tested by Galton in the 19th century [2,3]. The usual approach to assess cognitive function involves measuring the performance of individuals using a variety of variables—speed and accuracy, for example—on a range of psychometric tests. However, this traditional approach can be time consuming and inconvenient for participants and is accompanied by high economic costs relating to test design, administration and analysis [4]. Additionally, these assessments are typically undertaken one-on-one and require highly trained staff, placing further limitations on their accessibility. 

For large-scale studies, such intensive methods of assessment are not always feasible. Self-report assessments of cognitive abilities have been considered as a means of overcoming some of the practical and economic limitations associated with traditional assessments, as they are less expensive, more time efficient and easier to administer [5,6]. While a number of self-report cognitive assessment tools exist including, among others, the Prospective and Retrospective Memory Questionnaire (PRMQ) [7], the Cognitive Failures Questionnaire (CFQ) [8] and the Subjective Memory Complaints Questionnaire (SMCQ) [9], there are several limitations with their utility for cognitive research. For instance, most were designed as screening tools to identify cognitive extremes, such as dementia and are thus less concerned with assessing cognitive function within relatively healthy people. Additionally, these measures are highly ability-specific—usually focussing only on memory—and they do not provide a broad representation of other cognitive abilities [6]. Furthermore, the extent to which these tools accurately index cognitive function as measured by broad cognitive test batteries is seldom addressed by researchers [10,11].

Limited research has focused on the relationship between self-estimates of cognitive abilities and observed cognitive performance and the majority have utilised young-adult populations. Most studies, including meta-analyses (e.g., [12,13]), report weak to moderate correlations between self-estimated and performance-based cognitive abilities, with validity coefficients rarely exceeding *r* = 0.30 [14,15,16,17,18]. Thus, whilst the relationship is almost always significant, it is relatively weak, implying that self-report assessments of cognitive abilities measure something other than cognitive function. 

While a number of non-cognitive factors have been shown to influence self-estimates of cognitive abilities (e.g., gender and self-esteem), personality (most often operationalised using the Big Five Inventory) has received a considerable degree of attention [19,20]. Self-estimates of cognitive abilities have been repeatedly shown to relate positively to Extraversion and Openness to Experience and negatively to Neuroticism and Agreeableness [15,16,20,21,22,23,24]. Inflated self-estimates of cognitive abilities among those scoring high on Extraversion is considered a result of inherent overconfidence and assertiveness [20,22], while low self-estimates in those higher on Neuroticism reflects lower self-esteem and for Agreeableness, a degree of modesty [16]. Openness to Experience is the strongest personality correlate of intelligence and corresponding high self-estimates of abilities may be an accurate reflection of underlying status or alternatively, reflect their inclination towards enjoyment of intellectual activities [20]. In addition to the observed relationships between distinct personality traits and self-estimated cognitive abilities, multiple studies have shown that when combined, the Big Five personality factors predict substantial variance in self-report measures (up to 17%; [23,25]). Thus, it has been suggested that self-estimated cognitive abilities appear to depend more on personality than “actual” cognitive function itself [19]. 

The modest correlations observed between self-estimates of cognitive abilities and cognitive performance, combined with the influence of personality traits, suggests that people lack the ability to accurately estimate their underlying cognitive function. However, it has been argued that methodological limitations have compromised the validity of existing self-report tools [21,26,27]. Consequently, Jacobs and Roodenburg [4] developed a new tool: the Self-Report Measure of Cognitive Abilities (SRMCA). The SRMCA was designed to address limitations of other tools, by including multiple-items as opposed to single-items and phrasing questions in relative, rather than absolute terms; both of which have been found to significantly increase the validity of self-ratings [12]. A further advantage of the SRMCA is that its design was informed by one of the most influential and empirically supported theories of cognitive abilities currently available—the Cattell-Horn-Carroll (CHC) theory of cognitive abilities [4,28,29]. This theory proposes that human cognitive function encompasses a range of both broad and narrow cognitive abilities that are linearly independent. Accordingly, the SRMCA was designed to include multiple items that measure the distinct abilities of fluid reasoning, also referred to as fluid ability (Gf), comprehension-knowledge, also referred to as crystallised ability (Gc) and visual processing (Gv), which are central to general cognitive functioning [30,31]. 

Briefly, the Gf domain reflects an individual’s ability to form ideas, reason and solve problems that often involve unfamiliar concepts or procedures that cannot be performed by relying exclusively on previous learned habits, schemas and scripts [29,32]. The Gc domain represents the breadth and depth of a person’s acquired knowledge and skills [29]. This store of primarily verbal or language-based knowledge reflects those abilities that have been developed during education and general life experiences, such as the ability to answer factual questions and comprehend written and spoken language. The Gv domain represents an individual’s ability to generate, perceive, analyse and think with visual patterns and stimuli [29]. This domain influences one’s ability to perform tasks such as assembling puzzles and reading maps, charts and graphs [32]. 

To date, limited research has been published regarding the SRMCA. However, initial investigations using samples of young adults (mostly university students) have demonstrated interesting results. For example, Jacobs and Roodenburg [4] reported that the SRMCA adhered to the theorised three-factor structure, thus demonstrating discriminant validity for the different cognitive abilities measured across items. External validity of the SRMCA was also investigated, with analyses demonstrating that self-estimated Gc was significantly and moderately associated with performance on a measure of Gc and that self-estimated Gv correlated weakly with performance Gv. However, the self-estimated Gf domain was found to lack convergent validity, as it failed to correlate significantly with performance Gf [4]. 

The purpose of the present study was to further explore the utility of the SRMCA and expand on prior studies in two key ways. First, this study focussed on aged individuals. The reason for this reflects the increasing impact of cognitive decline in a dramatically ageing population [33,34]. Valid self-assessment tools would be beneficial in the field of cognitive ageing by facilitating large-scale studies and reducing the burden of formal cognitive assessments. Second, given the robust and demonstrated influence of personality traits on other self-report measures, this study considered the influence of personality on responses to the SRMCA. Distinct aims were to: (1) examine the factor structure of the SRMCA; (2) assess the reliability of the SRMCA domains over successive measurements; (3) explore the relationships between the SRMCA domains and performance on a battery of cognitive tasks; and (4) determine the impact of the Big Five personality traits on responses to the SRMCA. 

## 2. Method

### 2.1. Participants

Participants were *n* = 93 community dwelling older adults aged 52–82 years (*M* = 67.11, *SD* = 6.04). They were recruited from a pool of volunteers that had participated in a previous study of cognitive ageing [35] and who consented to being invited to participate in future studies. Inclusion criteria required participants to be between 50 and 85 years of age, to be proficient in the English language and to have normal (or corrected-to-normal) vision and hearing. As shown in Table 1, there were approximately equal numbers of males and females, English was the primary language and participants were relatively evenly distributed across measured education categories. 

### 2.2. Survey Materials

#### 2.2.1. Demographics

Participants were required to answer a series of demographic questions during the study questionnaire. The questions obtained participants’ age, gender, main language spoken and their obtained education levels (classified as Primary or High School qualification, Trade or other Certificate or Tertiary Qualification). 

#### 2.2.2. Self-Report Measure of Cognitive Abilities

The Self-Report Measure of Cognitive Abilities (SRMCA) [4] was included as a measure of self-estimated cognitive function. The SRMCA used herein was the 19-item measure outlined by Jacobs and Roodenburg [4]. These items measured the ability areas of fluid ability (Gf), crystallised ability (Gc) and visual processing (Gv). For each item of the SRMCA, participants rated how difficult or easy it is to perform particular types of cognitive tasks (e.g., understand written instructions) in comparison to “most people their age”. Response options were as follows: 1 (*extremely difficult*); 2 (*difficult*); 3 (*somewhat difficult*); 4 (*manageable*); 5 (*somewhat easy*); 6 (*easy*); and 7 (*extremely easy*). The items used herein are shown in Table 2.

#### 2.2.3. The Big Five Inventory

The Big Five Inventory (BFI) [36] was included as a measure of self-reported personality. The BFI used herein comprised of 44 items that provide domain scores for the five dimensions of the Big Five Model (i.e., Openness to Experience, Conscientiousness, Extraversion, Agreeableness and Neuroticism). For each item, participants indicated how much they agreed or disagreed with various statement (e.g., I see myself as someone who is outgoing and sociable) using a 5-point Likert scale. Response options were as follows: 1 (*disagree strongly*); 2 (*disagree a little*); 3 (*neither agree nor disagree*); 4 (*agree a little*); and 5 (*strongly agree*). Mean personality scores for each of the Big Five dimensions are shown in Table 1. 

### 2.3. Cognitive Assessment Battery

#### 2.3.1. Fluid Ability (Gf) Tasks

*Raven’s Progressive Matrices* (*RPM*). A modified version of RPM was used, which consisted of odd-numbered items from the original set [37]. Each trial presented a three-by-three array of symbols, with the bottom right hand symbol missing. Participants were instructed to choose from options given below the matrix which symbol logically completed the matrix. Twenty items were administered within a ten-minute time limit that became progressively more difficult. Two practice trials of easy items were completed for familiarisation purposes. The outcome measure was the total number of items correctly completed. 

*Letter Sets* (*LS*). This task was derived from the Educational Testing Services (ETS) Kit of Factor-Referenced Tests [38]. Stimuli consisted of a series of five, four-letter items which followed a predetermined alphabetical ordering. Participants were required to determine the rule or pattern linking four of the items and cross out the item not fitting the rule. This task included fifteen items which were administered within a seven-minute time limit. Two practice trials of easy items were completed for familiarisation purposes. The outcome measure was the total number of items correctly completed. 

#### 2.3.2. Crystallised Ability (Gc) Tasks

*Vocabulary* (*VOC*). This multiple-choice task involved 21 vocabulary test items from the ETS Kit of Factor-Referenced Tests [38]. For each trial, a test word was presented together with five answer words. Participants were required to mouse-click the answer word that had similar meaning to the test word (e.g., jovial/jolly). No practice trials were given and a four-minute time limit was imposed. The outcome measure was the total number of items correctly completed. 

*Spot the Word* (*STW*). This task involved the presentation of sixty items comprising one word and one non-word [39]. Participants indicated, using the computer mouse, which word in each pair was a real word (e.g., xylophone/flonty). Words were presented centrally on the computer screen, one above the other. No practice trials were given and a five-minute time limit was imposed. The outcome measure was the total number of items correctly completed. 

### 2.4. Procedure 

Ethics approval for this study was obtained from the University of Adelaide Human Research Ethics Committee and the CSIRO Human Research Ethics Committee. Willing participants from a previous study of cognitive abilities (data collected in September 2014; see [35]) were emailed an invitation to participate in the present study in May 2015. The email briefly outlined the nature of the study and included a link to an online survey and the online information sheet. After viewing the information sheet, participants were asked to indicate whether they wished to participate and, if so, provided online consent. This consent included permission to match their performance on the cognitive battery described herein but collected ~8-months prior with data obtained during this study. Participants then completed a 15-min questionnaire which included the SRMCA, the Big Five Inventory and some general demographic questions. Approximately eight weeks after completing the first questionnaire, *n* = 53 participants completed a second online questionnaire in order to obtain SRMCA reliability estimates. 

### 2.5. Power and Statistical Analyses 

Power analyses were conducted prior to data collection to determine the minimum number of participants required using the G*Power software program [40]. For bivariate correlations, a minimum number of 81 participants was required to identify weak relationships as low as *r* = 0.35, assuming α = 0.05 and β = 0.90. For linear regressions with up to seven predictors (2 × cognitive; 5 × personality), a minimum number of 80 participants was required to identify a moderate multiple correlation of *r* = 0.45, assuming α = 0.05 and β = 0.80. For the paired-samples *t*-tests, a minimum number of 67 participants was required, assuming a small effect size (Cohen’s *d* = 0.30), α = 0.05, β = 0.90 and a weak correlation between the measures (*r* = 0.30). Finally, for factor analysis of the SRMCA, a minimum participant to item ratio of 4:1 which is considered adequate given the reported high communalities between the SRMCA items [41] required a total sample of 76 participants. 

All statistical procedures were performed using the Statistical Package for the Social Sciences (SPSS) software—Version 23, with a required critical alpha level of *p* < 0.05. Data for the cognitive tests were collated and assessed for missing values and outliers (i.e., ± 3 *SD*). These values (19 in total, ~1%) were confirmed to be missing at random and replaced using the Multiple Imputation procedure in SPSS. Then, factor scores were generated by extracting a single underlying Principal Component and saving the factor score using the regression method (e.g., Matrices and Letter Sets for Gf, Spot-the-Word and Vocabulary for Gc, all tasks for G). The IQ distribution of this sample was then estimated based on performance on Spot-the-Word and existing normative data for this age group [42]. Standardised distributions of the cognitive performance factors (Gf, Gc and G) were then transformed to IQ-equivalents based on the estimated IQ of this sample to aid interpretation of the analyses relating to self-estimated ability. 

## 3. Results

### 3.1. Descriptive Statistics for the SRMCA

Descriptive statistics and item reliabilities for the SRMCA are displayed in Table 2. As can be seen, item means were comparable across time points and participants mostly rated the various cognitive activities questioned in the SRMCA items as “easy”. Item reliabilities were generally moderate to strong across time points. However, two items (one Gf and one Gv) demonstrated unacceptably low reliabilities (<0.50). Cronbach’s alphas were calculated for the SRMCA at each time point and demonstrated acceptable levels of internal consistency (Time 1 α = 0.95; Time 2 α = 0.95). Alpha reliabilities for item groups at Time 1 for Gf, Gc and Gv were α = 0.89, α = 0.90 and α = 0.92 and at Time 2 were α = 0.91, α = 0.89 and α = 0.92, respectively.

### 3.2. Factor Structure of the SRMCA

The latent structure of the SRMCA was examined using exploratory factor analysis (EFA). The Kaiser-Meyer-Olkin (KMO) measure of sampling adequacy index was 0.92 and KMO values for individual items in the diagonals of the anti-image matrix were ≥0.84. Bartlett’s test of sphericity (χ^2^(171) = 1374.18, *p* < 0.001) also indicated that the SRMCA data were appropriate for EFA. Consistent with prior work by Jacobs and Roodenburg [4], initial analysis utilised Principal Axis Factoring (PAF) with Direct Oblimin (oblique) rotation and factors with eigenvalues ≥1 were extracted. This solution (see Table 2) resulted in two factors that accounted for 64.35% of the variance. This two-factor solution appeared to comprise of a Gf/Gv factor (F1) and a second factor defined primarily by Gc items (F2). Furthermore, several items were moderately cross-loaded, making interpretation of this solution difficult. Given the theorised three-factor structure of the SRMCA, the scree plot was inspected which revealed an inflexion commencing at the third component that had an eigenvalue of 0.98. Therefore, an additional PAF with three-factors was specified. The three factors accounted for 69.48% of the variance. The three-factor solution (see Table 2) improved clarity to some degree and appeared similar to the theoretical model. However, many Gv items cross-loaded together with Gf items, or did not load as expected. 

### 3.3. Reliability of the SRMCA Factors

The underlying solution did not conform directly to the theorised structure. However, there is significant overlap between the abilities of Gf and Gv and they can therefore be difficult to separate from one another [28,43]. In light of this, composite mean scores based on the original theorised model were calculated using all relevant items. A broad SRMCA G domain representing overall cognitive function was also calculated by averaging responses across all items. Test-retest reliability estimates and correlations between SRMCA domains are shown in Table 3. The reliability of the SRMCA domains was generally strong and although still adequate, was lowest for Gf. The correlations between the SRMCA domains were all strong and statistically significant, with the strength of these indicating high commonality across self-estimated ability domains.

### 3.4. Relationships between the SRMCA, Cognitive Performance and Personality

Correlations between the SRMCA domains, cognitive performance factors and personality are shown in Table 3. As can be seen, all of the SRMCA domains were significantly and positively correlated with performance Gc, with the strongest correlation between self-estimated and performance Gc. Contrary to expectations, neither self-estimated Gf nor Gv was associated with performance Gf. However, all SMRCA domains shared significant variance with broad cognitive performance G. With regard to personality traits, Extraversion was significantly and positively correlated with all SRMCA domains, as expected. Somewhat stronger positive correlations were present between Openness to Experience and SRMCA domains, whilst Neuroticism was inversely related to self-estimated Gf and G domains. 

Stepwise regression was performed to explore the influence of demographics (age and sex), personality factors and cognitive performance factors on SRMCA scores. All variables were entered at the first step, with Backward elimination used to arrive at the combination of best predictors. The solutions for the first and final steps are provided in Table 4. In terms of demographics, males had higher scores on the self-estimated domains than females for self-estimated Gf, Gv and G, whilst age was not related. Of the cognitive performance factors, Gc was the only significant predictor of SRMCA domains. It was most strongly related to the corresponding self-estimated Gc domain, as expected and weakest with the self-estimated Gv domain. In terms of personality, Openness to Experience was a relatively constant predictor of all SMRCA domains yet contributed least to self-estimated Gc. In addition to this, Neuroticism was significantly inversely related to self-estimated Gf and G domains, whilst Extraversion was positively related to self-estimated Gc. To confirm reliability of these findings, the regression models were reassessed using forward elimination and the findings were entirely consistent.

### 3.5. Demographic and Personality Biases

A comparison between self-estimated versus actual cognitive performance was undertaken by converting SRMCA domain scores to IQ equivalents based on the estimated IQ distribution of the sample (see Power and Statistical Analyses section). Comparison of means across corresponding scores for each of Gf, Gc and G, was then undertaken for demographic groups and also personality groups, which were determined via a median split of each personality dimension. Analysis of self-estimated Gv was not performed because of the absence of a true corresponding performance Gv factor.

Results of these analyses are presented in Table 5. Regarding demographics, those with tertiary education underestimated Gf by ~5-IQ points. Females significantly underestimated Gc and G by ~6-IQ points, with males overestimating Gc and G by a similar amount. The younger group (≤67 years) underestimated Gf, whilst the older group overestimated Gf. Results in relation to personality dimensions were varied but all in the expected direction. Those higher in Extraversion overestimated Gc by ~7-IQ points whilst those lower in this trait underestimated G by ~5-IQ points. Individuals higher in Neuroticism underestimated Gc, Gf and G by as much as ~7-IQ points. For Openness to Experience, higher individuals significantly overestimated Gf by ~9-IQ points and G by ~7-IQ points.

## 4. Discussion

The aim of this study was to examine whether the recently published Self-Report Measure of Cognitive Abilities (SRMCA) [4] is a reliable and valid measure of cognitive function in a sample of community-dwelling older adults. To achieve this, we examined the factor structure of the SRMCA, the reliability of SRMCA domains over successive measurements, the relationships between the SRMCA domains and test-based cognitive performance and the influence of the Big Five personality traits on participants’ responses. 

Though psychometric scales can sometimes be reliable without being valid, reliability is a necessary condition for validity [44]. The internal consistency for the SRMCA items was relatively high for both time points for the Gf, Gc and Gv domains. This is consistent with the high alpha reliabilities reported by Jacobs and Roodenburg [4], thus providing further support for the internal reliability of the SRMCA. Moreover, individual item reliabilities were generally acceptable but it was apparent that participant responses were consistently in the higher-end of the response scale. Specifically, the overall mean response was ~5 corresponding to the anchor of “somewhat easy.” Thus, it would appear that, despite the age of this sample (67 years, on average), participants did not appear to subjectively rate the broad range of cognitive activities questioned across the items as overly difficult. 

In terms of test-retest reliability, this study explored the stability of responses approximately two months apart. The SRMCA domains (particularly Gc and Gv) demonstrated adequate reliability when completed eight weeks later. However, the reliability of Gf was notably lower and did not meet the minimum recommended coefficient of 0.70 [45]. Nevertheless, the SRMCA was not designed to be used as a clinical tool and the reliability seen here suggests it might still be suitable for research purposes. 

Consistent with prior research concerning the development of the SRMCA, it was anticipated that a three-factor solution would describe the data. However, the theorised three-factor structure did not naturally emerge. Initial analysis revealed a two-factor solution consisting of Gc and a second factor comprised of Gf and Gv items. This is inconsistent with findings by Jacobs and Roodenburg [4] who demonstrated that the SRMCA accurately replicated the theorised three-factor structure. Extension of the solution to include three factors did provide some limited evidence of three underlying factors representing Gc, Gf and Gv. However, there remained significant overlap with many Gv items continuing to load strongly on Gf. Thus, while participants appeared to be able to distinguish Gc activities, as evidenced by the emergence of a relatively clear Gc in both solutions, they appeared to have particular trouble differentiating between Gf and Gv domains. This might be due to the fact that Gf and Gv are typically highly correlated [28,43] and that their corresponding SRMCA items are similar in the sense that both are concerned with efficient and effective processing of incoming information [4,46]. On the contrary, the very strong correlations present between the SRMCA domains suggests that although participants appeared “somewhat” able to distinguish between domains, participant responses mostly reflected general cognitive ability, rather than discrete types of abilities [4,12,27]. This latter interpretation is supported by the high commonality across SRMCA domains, reflected in the strong correlations.

In addition, it is possible that the inconsistent findings could also be due to methodological limitations, rather than a result of the instrument and/or participants. Specifically, the relatively modest sample size used herein, in comparison to the larger sample utilised by Jacobs and Roodenburg [1], may have compromised the solution achieved and thus future studies with larger samples may obtain different results. With this said, it has been suggested that it is not the overall sample size that it of concern but rather, the ratio of participants to items. The participant to item ratio in the present study was 5:1 and although it has been argued that smaller ratios are acceptable when commonalities between items are high [44], some authors advise that ratios of up to 20:1 are required [47].

The degree to which the domains assessed in the SRMCA relate to cognitive performance measures was variable. Self-estimated Gc was significantly and positively associated with performance Gc, consistent with findings by Jacobs and Roodenburg [4]. Of particular interest is that correlations with performance Gc in this study exceeded the frequently cited limit of *r* = 0.30 (e.g., [12,13,17]), as well as that (*r* = 0.37) reported by Jacobs and Roodenburg [4] for young adults. Contrary to this, self-estimated Gf and Gv domains did not significantly correlate with the performance Gf factor, as expected. Instead, they correlated significantly only with Gc as well as the overall G factor. This lack of a significant relationship between self-estimated Gf and performance Gf is consistent with previous findings regarding the SRMCA [4] but inconsistent with other research concerning the relationship between self-estimated Gf and performance Gf (e.g., [18,21,24,27]).

There are many plausible reasons for the higher correlations observed between self-estimated and performance Gc. For example, participants in the present study had previous experience with the cognitive assessment battery, which has been suggested to be particularly beneficial for increasing the validity of self-estimates in older adults [12,48]. Specifically, given that participants completed the test battery prior to the SRMCA by ~8-months, they may have had improved insight into their actual cognitive performance skills through this experience. Furthermore, crystallised abilities improve as a result of increased life experiences and opportunities to develop new knowledge [49,50]. It is therefore probable that the participants in this study had increased insight into their Gc (such as through education) and were better able to judge this in comparison to previous studies involving students/younger adults. 

The significant correlations between performance Gc and self-estimated Gf and Gv are likely due to there being considerably less opportunities available for individuals to receive feedback regarding their own and other’s performance on Gf and Gv activities. As a result, it is probable that participants had much more difficulty in determining their actual level of performance for these abilities and thus based their evaluations of Gf and Gv on the better known Gc. In addition, Gf and Gv are subject to age-related changes and thus it may be more difficult for older adults to have insight into these particular abilities [51].

In light of prior research showing relationships between personality and one’s ability to self-estimate their intelligence, the influence of personality traits on the SRMCA was examined and this study appears to be the first to report on these associations. Despite the fact that the SRMCA was designed to overcome limitations of prior tools by including multiple items, phrasing questions in relative terms and being based on a comparatively more well-validated theory of cognitive abilities, it was still moderately influenced by personality traits. Specifically, those higher on Extraversion and in particular, Openness to Experience, significantly over-estimated their cognitive abilities, while those higher on Neuroticism were found to under-estimate them. In fact, the regression models reported herein indicate that self-estimated Gf and Gv more strongly reflect one’s personality than their actual cognitive performance ability. It is likely that responses to such items at least partly reflect an individual’s confidence or perceived life experiences. These results are generally consistent with previous literature regarding other self-report tools and suggest that, despite its methodological advancements, the SRMCA does not appear to have overcome this personality bias (e.g., [15,16,19,20,21,22,23,24]).

While the current study addressed an important gap in the literature by examining the efficacy of a self-report measure of cognitive abilities in older adults and in particular, being the first study to investigate the test-retest reliability of the SRMCA and its relationship with personality, a number of limitations must be considered. First, only two measures of each of Gf and Gc were included. A more diverse battery of tasks may therefore lead to different conclusions insofar as a larger battery might quantify increased individual differences in these broad abilities. Similarly, despite evidence of a strong overlap between Gf and Gv [28,43], the inclusion of tasks purported to measure Gv specifically would permit a more thorough interpretation of the relationship between self-estimated Gv and performance measures. The characteristics of participants in this study should also be considered a potential limitation. Despite the fact they were relatively evenly distributed in terms of education and sex, they were at least intrinsically motivated to participate in the present study regarding cognitive function. Their attendance at the testing laboratory is likely indicative of higher Openness to Experience and potentially, Extraversion, than usual. This may have contributed to the strong personality bias apparent herein, particularly for Openness and self-estimated ability. Furthermore, due to the modest sample size, the present study did not have sufficient power to consider the moderating effects of sex and age on self-estimated cognitive ability. Future studies with a larger sample should endeavour to explore this.

Jacobs and Roodenburg [4] anticipated that a successful self-report measure of cognitive abilities could result in beneficial outcomes for practicing psychologists and their clients through a reduction in the amount of time and money required to assess cognitive abilities. Thus, this study aimed to further consider the SRMCA in an older population in order to transfer these practical and economic efficiencies to cognitive research more generally. The present findings bring in to question whether the SRMCA is a useful tool for this purpose and indeed, whether it is an improvement on other self-report measures of cognitive ability. While the SRMCA appeared to be generally reliable over a short period of time, the Gf and Gv domains failed to correlate with observed cognitive performance. Additionally, the SRMCA did not adequately replicate the theorised three-factor solution and individual response to this measure were significantly influenced by personality. 

Future studies could focus on expanding the application of the SRMCA by considering its efficacy as an informant-based evaluation of cognitive function. Having one’s significant other judge their cognitive ability relative to peers of the same age may improve the validity of this tool, particularly in terms of removing the influence of personality on this measure. Overall, given that the SRMCA is a new self-report measure of cognitive functioning, it is evident that much more research is needed. Future research should consider whether the SRMCA has any other clinical utility other than cognitive function. For example, discrepancies between self-assessments and objective measures may point to failures in metacognitive efficiency. However, if similar findings are obtained upon replication with a larger, more diverse sample of participants, resources and funding may be better directed towards traditional cognitive testing procedures.

## Figures and Tables

**Table 1:** Sample characteristics and mean personality scores.

Mean (*SD*)	
Age	67.1 (6.0)
IQ	102.1 (17.8)
Extraversion	3.4 (0.8)
Agreeableness	4.0 (0.5)
Conscientiousness	4.1 (0.5)
Neuroticism	2.4 (0.8)
Openness	3.7 (0.5)
*n* (%)	
Male	47 (50.5%)
English main language	83 (89.2%)
Born in Australia	73 (78.5%)
Education—School only	28 (30.1%)
Education—Trade/Certificate	30 (32.3%)
Education—Tertiary Educated	35 (37.6%)

**Table 2:** Descriptive statistics for SRMCA items and item loadings resulting from Principal Axis Factoring.

SRMCA Items	Descriptive Statistics					PAF Solution 2 *^a^*		PAF Solution 2 *^b^*		
	T1 *M*	T1 *SD*	T2 *M*	T2 *SD*	*r*	F1	F2	F1	F2	F3
Gf. Anticipate outcomes	5.27	0.96	5.09	0.88	0.57	0.26	0.56	0.55	0.32	
Gf. Come up with a solution to a problem never experienced before	4.77	1.13	4.64	0.90	0.55	0.62		0.70		
Gf. Come up with a strategy to solve a difficult problem	4.75	0.95	4.70	1.05	0.78	0.60	0.29	0.63		
Gf. Look at a problem from multiple perspectives	5.00	0.93	5.02	0.93	0.36	0.57		0.67		
Gf. Use information I have learnt previously in a new context	4.91	0.92	4.77	0.93	0.55	0.29	0.57	0.80		
Gc. Convey precisely what I am trying to say	5.12	1.03	5.00	1.00	0.71		0.71		0.65	
Gc. Demonstrate my word knowledge	5.31	1.00	5.19	1.06	0.67		0.70		0.56	
Gc. Display the extent of my general knowledge	5.40	0.97	5.21	0.93	0.62		0.88	0.38	0.64	
Gc. Express a large vocabulary	5.10	1.14	5.02	1.15	0.74		0.95		0.95	
Gc. Think of the correct name of an object	4.78	1.08	4.70	0.99	0.72		0.71		0.69	
Gc. Understand written instructions	5.30	0.92	5.15	0.97	0.59		0.57	0.47	0.35	
Gv. Complete games that require visual skills	5.28	1.03	5.17	0.89	0.63	0.32	0.39	0.45		
Gv. Determine if furniture will fit in a room just by visualising it	4.89	1.17	4.83	1.28	0.75	0.79				0.78
Gv. Follow visual diagrams that come with “assemble yourself” products	4.96	1.24	4.92	1.27	0.78	0.67		0.70		
Gv. Imagine how an object will look when it is completed	4.92	0.99	4.85	1.26	0.76	0.92				0.75
Gv. Imagine what an object would look like from a different angle	4.63	0.98	4.60	1.21	0.65	.94		0.47		0.54
Gv. Mentally rotate three-dimensional images in my mind	4.48	1.14	4.43	1.05	0.42	0.70		0.66		
Gv. Understand information presented in a visual format	5.34	0.99	5.17	1.03	0.50	0.52	0.35	0.60		
Gv. Visually estimate if something will fit	5.29	1.10	5.42	1.29	0.65	0.85				0.76

Note: T1 = Time 1 (*n* = 93); T2 = Time 2 (*n* = 53). Item loadings ≤0.25 suppressed for clarity. *^a^* Factors derived based on Eigenvalues ≥1. *^b^* Specified three factor solution.

**Table 3:** Correlations between SRMCA domains, cognitive performance factors and personality traits.

SRMCA Domain	Retest Reliability	Self-Report			Cognitive Performance			Personality				
	*r ^a^*	Gc	Gv	G	Gc	Gf	G	E	A	C	N	O
SRMCA Gf	0.67 **	0.74 **	0.79 **	0.92 **	0.28 **	0.16	0.26 *	0.30 **	0.17	0.25 *	−0.30 **	0.53 **
SRMCA Gc	0.78 **	-	0.64 **	0.86 **	0.51 **	0.26 *	0.46 **	0.27 **	0.20	0.08	−0.15	0.41 **
SRMCA Gv	0.79 **	-	-	0.93 **	0.23 *	0.14	0.23 *	0.21 *	0.07	0.19 *	−0.14	0.40 **
SRMCA G	0.75 **	-	-	-	0.37 **	0.20	0.34 **	0.28 **	0.15	0.19 *	−0.21 *	0.48 **

Note: E = Extraversion; A = Agreeableness; C = Conscientiousness; N = Neuroticism; O = Openness. *^a^* Correlation between SRMCA composite factor scores at Time 1 and Time 2. * *p* < 0.05, ** *p* < 0.05.

**Table 4:** First and final models arising from backward stepwise regression predicting SRMCA domains with demographics, cognitive performance and personality traits.

D.V.	SRMCA Gf		SRMCA Gc		SRMCA Gv		SRMCA G	
Predictor	S1	FM^5^	S1	FM^5^	S1	FM^6^	S1	FM^5^
Model R	0.68	0.67	0.66	0.64	0.59	0.55	0.65	0.64
Model R^2^_adj_	0.41	0.43	0.39	0.39	0.27	0.27	0.36	0.38
Age	−0.01		−0.01		0.16		0.07	
Sex	0.25 **	0.22 **	−0.04		0.29 **	0.29 **	0.20 *	0.18 *
Performance Gc	0.29 **	−0.30 **	−0.49 ***	−0.49 ***	0.20 *^a^*	0.26 **	−0.35 ***	−0.38 ***
Performance Gf	0.08		−0.04		−0.17		−0.12	
Extraversion	0.07		−0.15	−0.22 *	−0.09		−0.12	
Agreeableness	−0.07		−0.01		−0.04		−0.04	
Conscientiousness	0.07		−0.11		−0.14		0.05	
Neuroticism	−0.31 **	−0.33 ***	−0.17		−0.14	−0.19 *^a^*	−0.21 *^a^*	−0.25 **
Openness	0.40 ***	−0.44 ***	−0.30 **	−0.25 **	−0.24 *	0.35 ***	−0.33 **	−0.40 ***

Note: D.V. = Dependent Variable. S1 = Step 1. FM*^n^* = Final Model with *n* denoting total number of steps. *^a^ p* < 0.10, * *p* < 0.05, ** *p* < 0.01, *** *p* < 0.01.

**Table 5:** Means, (SDs) and differences between IQ-equivalent SRMCA (SR) and cognitive performance (COG) scores across demographics and personality types.

Variable	Category	Gf		∆	Gc		∆	G		∆
		SR	COG		SR	COG		SR	COG	
Ed	High School	99.48	95.83		100.57	101.06		100.86	97.87	
		(19.18)	(16.2)		(19.95)	(16.97)		(20.12)	(17.28)	
	Certificate	101.65	95.88		99.85	99.48		100.33	96.98	
		(16.38)	(17.97)		(17.15)	(18.85)		(15.8)	(19.28)	
	Tertiary	104.66	112.53	↓ *	105.33	105.26		104.69	109.95	
		(18.13)	(13.11)		(16.68)	(17.17)		(17.81)	(13.36)	
Sex	Females	99.20	106.46	↓ *	104.17	104.24		100.03	106.10	↓ *
		(19.69)	(18.74)		(19.15)	(16.51)		(19.9)	(17.69)	
	Males	104.99	97.89	↑ *	100.13	100.07		104.19	98.24	↑ *
		(15.53)	(15.36)		(16.44)	(18.66)		(15.53)	(16.71)	
Age	≤67	102.21	109.08	↓ *	103.49	100.55		101.92	105.43	
		(16.43)	(18.46)		(18.00)	(18.90)		(17.64)	(19.19)	
	68+	102.04	94.72	↑ *	100.67	103.82		102.36	98.61	
		(19.44)	(13.11)		(17.78)	(16.27)		(18.27)	(15.06)	
E	Lower	97.31	102.02		97.68	104.04	↓ **	97.77	103.33	↓ *
		(17.13)	(17.85)		(16.72)	(19.18)		(16.96)	(18.16)	
	Higher	107.50	102.25		107.09	100.00	↑ **	106.98	100.80	
		(17.27)	(17.44)		(17.95)	(15.74)		(17.75)	(16.97)	
A	Lower	99.85	103.76		98.97	101.13		100.15	102.62	
		(18.21)	(17.91)		(17.76)	(19.91)		(17.17)	(18.07)	
	Higher	104.01	100.79		104.73	102.96		103.75	101.73	
		(17.51)	(17.33)		(17.68)	(15.72)		(18.40)	(17.29)	
C	Lower	98.43	101.75		101.62	102.94		99.20	102.26	
		(16.77)	(16.77)		(18.45)	(16.85)		(16.41)	(17.18)	
	Higher	105.60	102.48		102.61	101.36		104.87	102.01	
		(18.31)	(18.44)		(17.46)	(18.53)		(18.86)	(18.09)	
N	Lower	106.27	100.50		104.41	99.03	↑ *	105.41	99.19	↑ *
		(17.65)	(18.49)		(18.43)	(17.62)		(18.10)	(18.03)	
	Higher	97.51	103.94	↓ *	99.59	105.58	↓ *	98.47	105.41	↓ **
		(17.11)	(16.48)		(17.04)	(17.25)		(17.04)	(16.61)	
O	Lower	94.79	101.69	↓ *	97.11	100.94		95.75	101.28	↓ *
		(15.42)	(18.33)		(16.85)	(17.92)		(15.97)	(18.05)	
	Higher	111.44	102.69	↑ *	108.49	103.64		110.21	103.20	↑ *
		(16.46)	(16.74)		(17.23)	(17.42)		(16.97)	(17.07)	

Note: Ed = Education; E = Extraversion; A = Agreeableness; C = Conscientiousness; N = Neuroticism; O = Openness. ∆ Significant difference estimated with paired-samples *t*-test. ↑ overestimated one’s ability; ↓ underestimated one’s ability. Comparisons of self-estimated Gv were not performed given the absence of a corresponding cognitive performance factor. * *p* < 0.05, ** *p* < 0.01.
